# Seeing Flames, Perceiving Quantity: Approximations of Fire Intensity Across Development

**DOI:** 10.3390/bs15101397

**Published:** 2025-10-15

**Authors:** Justin W. Bonny

**Affiliations:** Department of Psychology, Morgan State University, Baltimore, MD 21251, USA; justin.bonny@morgan.edu

**Keywords:** general magnitude system, fire intensity, approximate number system, cognitive development, human behavior in fire

## Abstract

Between three and six years of age, children become better able to detect smaller differences in non-symbolic visual quantities. This includes judging which set of objects are greater in number and area. These findings suggest that the underlying approximate magnitude representations, which facilitate the estimation of these quantities, become more precise with age into adulthood. Such parallels in developmental trends raise questions about the extent to which they are observed across different non-symbolic quantities. The present study addressed this question using a novel quantity: visual fire intensity. Like number and area, fire intensity can be estimated using visual cues and has real-world implications. However, it is unclear whether young children accurately compare fire intensities and if there are age-related differences in performance. The present study investigated the developmental trend of young children’s visual perception of fire intensity. Over 70 three- to six-year-olds completed a comparison task where they judged which of two train engines had a more intense fire. Based on non-symbolic quantity research, the ratio (relative difference) between the intensity of two simulated fires was varied across trials to be smaller versus larger. Significant ratio and age effects were observed: children were more likely to select the train with the greater-intensity fire as being ‘more’ the larger the ratio and the older the child. These results suggest that young children are sensitive to differences in fire intensity using visual cues and have increasingly precise estimates by six years of age. This developmental pattern indicates that approximate magnitude representations support perceptions of ecologically relevant, dynamic quantities such as fire intensity.

## 1. Introduction

People frequently make ordinal judgments about non-symbolic quantities in the real-world, including ‘how much’ food is available and ‘how many’ people are in the classroom. Detecting the ordinal relations between non-symbolic quantities is supported by approximate representations of magnitude (Cantlon et al., 2009). These analog representations are noisy mental estimates of quantities with the level of representational precision increasing with age (Halberda & Odic, 2015). Multiple behavioral and neural characteristics of approximate magnitude representations have been described for several quantities, most frequently number, spatial extent, and time (Brannon, 2002; Dehaene & Brannon, 2010; Odic et al., 2013). These characteristics have been observed early in development, including some studies reporting evidence within three months after birth (Izard et al., 2009; Xu et al., 2005). Children become better able to detect smaller differences in visual non-symbolic quantities, including number, size, and density, from three to six years of age (Bonny & Lourenco, 2013; Halberda & Feigenson, 2008; Odic, 2018; Odic et al., 2013). These improvements continue into adulthood (Halberda et al., 2012; Libertus et al., 2011). The degree to which approximate magnitude representations of additional quantities present in the environment share cognitive mechanisms across development remain to be explored. In addition, non-symbolic quantities can be static, remaining consistent in form and value, or dynamic, varying with time. A dynamic quantity that has important real-world implications is the intensity of fire. Models of human behavior in building fires emphasize the importance of detecting relevant cues. Occupants must perceive these cues in order to evaluate an incipient fire and decide on further action (Kuligowski, 2013; Kuligowski & Mileti, 2009). These cues can present in multiple different sensory modalities and vary as time elapses since ignition. As such, fire intensity provides an opportunity to evaluate the perception of a dynamic non-symbolic amount as a comparison to prior research with number, size, and density. Whether young children can use visual cues to judge fire intensity, and whether age-related differences similar to other quantities are observed, has yet to be examined. The present study investigated changes in the precision of fire intensity perception during early childhood.

### 1.1. Developmental Trends in Non-Symbolic Quantity Perception

As early as in infancy, children can perceive differences in non-symbolic quantities. Values of these quantities, including number, size, and duration, are displayed non-symbolically, without the use of cultural constructions (e.g., Arabic numerals) and in such a manner to prevent older children and adults from using precise measurement (e.g., no counting; Cantlon et al., 2009). Non-symbolic quantities have been most commonly studied using visual displays (e.g., image with 18 dots to present number) with several values presented to participants. A reliable pattern of performance when comparing two values of non-symbolic quantities is the ratio effect, which is a decrease in performance as the two values being compared become closer. Ratio effects have been observed with visual displays of number, spatial extent, and duration in behavioral measures collected from several types of experimental methods; this includes explicit ordinal judgments (e.g., accuracy and response time when making “which is more” judgments) made by children and adults (Lourenco et al., 2012; Odic et al., 2013). The presence of ratio effects across the lifespan points to developmental continuity in underlying representations of non-symbolic quantity (Feigenson et al., 2004; Starr & Brannon, 2015). These analog representations are characterized as approximate Gaussian distributions along a mental more-versus-less continuum, centered at the corresponding value of the non-symbolic quantity and the width corresponding to the level of representational noise (Feigenson et al., 2004). This level of noise contributes to overlap between representations of quantities that are close in value, producing the ratio effect (e.g., number, DeWind et al., 2015). For example, individuals are more likely to correctly identify which of two non-symbolic quantities is greater in value when the corresponding mental representations overlap to a lesser extent. Thus, ratio effects have been used as indicators of approximate magnitude representations of non-symbolic quantity.

Developmental changes in the ratio effect have been attributed to the increasing precision of approximate magnitude representations. Weber fractions (*w*s) are commonly used to quantify the amount of noise in the Gaussian distributions of approximate magnitude representations (Halberda & Odic, 2015). Individuals that have a lower *w* have less overlap in corresponding mental representations and are more likely to be able to distinguish between quantities closer in value. Prior research has compared *w* values across age groups as well as between different types of quantities. Developmentally, studies have observed a rapid improvement in *w* values between infancy and early childhood, across different types of quantities. For non-symbolic number, whereas 6-month-old infants can reliably distinguish between 12 versus 6 dots (*w* = 1.0; Xu et al., 2005), this improves to 12 versus 8 dots for 3-year-olds (*w* = 0.525) and 7 versus 6 dots for 6-year-olds (*w* = 0.179; Halberda & Feigenson, 2008). Similar developmental trends have been observed with length, area and duration judgments, as well as with other set-based quantities such as density (number of dots per unit area; Odic, 2018). This body of research indicates that non-symbolic quantities that utilize approximate magnitude representations should display similar age-related improvements in ratio effects and *w* values, with the most significant improvements occurring in early childhood.

Similarities in the developmental trajectories, among other evidence, have been the subject of theoretical debates on the nature of approximate representations for different non-symbolic quantities. This has focused on whether the corresponding analog representations for quantities such as number, size, and time are supported by shared versus distinct cognitive processes (c.f. Bueti & Walsh, 2009; Odic, 2018). The shared view proposes that the source of similar behavioral and neuroimaging characteristics is a shared process to form a common analog representation for non-symbolic quantities (Walsh, 2003). In contrast, the distinct view posits that separate processes correspond to different non-symbolic quantities, with similar characteristics such as ratio effects due to comparative mechanisms used to detect ordinal relations between analog representations (Anobile et al., 2016; Odic, 2018). As it pertains to developmental trends, evidence suggesting distinct representations includes different rates of age-related changes in comparison task performance for number and area judgments of 7- to 11-year-olds (Yan et al., 2024) and estimates of the ages at which precision reaches peak levels being earlier versus later ages for different quantities (Odic, 2018). Overall, this suggests that comparing the developmental trends in corresponding *w* values can be used to investigate the extent to which non-symbolic quantities share underlying processes.

### 1.2. Representation of a Novel Non-Symbolic Quantity: Fire Intensity

Decision-making in real-world contexts frequently depends on perceiving and estimating quantities in situated environments over a period of time. Although ratio effects of number and spatial extent are typically studied using artificial stimuli (e.g., dots and squares), several studies have examined approximate representations using ecologically relevant stimuli. This includes food items with children (e.g., cereal, vanMarle & Wynn, 2011), batteries (Gordon, 2004), objects within photographs of real-world scenes (e.g., animals, produce, Odic & Oppenheimer, 2023), and slopes of terrain (e.g., hills, Proffitt et al., 1995). The use of stimuli that are closer to how things appear in real environments aligns with calls to include more ecologically relevant stimuli in psychological science (Pooja et al., 2024; Schmuckler, 2001). For example, evolutionary theories of approximate number representations are based on the importance of situated estimates of number to animal survival including foraging, navigation, predator avoidance, and cooperative hunting (Nieder, 2020). Several types of ecologically relevant and dynamic quantities are commonly encountered and have evolutionary importance in survival and thriving. These real-world instantiations of non-symbolic quantities include water flow in rivers, vehicle speed, and wind direction, among others. Based on psychophysics and perception research (Stevens & Galanter, 1957), such quantities are mentally represented as analog distributions. However, whether representations of dynamic, ecological quantities display similar characteristics to those observed for number, area, and time remains to be determined. Investigating ratio effects and Weber fractions can provide insight into whether quantities across different ecologically relevant categories are part of a broader approximate magnitude system with potentially shared processes.

In terms of evolutionary and cultural importance, a dynamic, ecologically relevant quantity is fire intensity. Compared to other dynamic real-world quantities, fire is unique in that it permeates several aspects of daily life, being used in multiple tasks and settings. Fire can be used for multiple productive functions, including cooking and social gatherings (Wrangham & Carmody, 2010). However, fire can be destructive when uncontrolled, leading to the loss of property and life (Hall, 2023). The intensity of fire is directly related to maintaining fire as a productive, rather than destructive, force. A challenge to maintaining the intensity of fires is that the quantity is typically dynamic: perceptual cues can fluctuate with time, requiring a period to estimate the value. Although laminar flames can be encountered in settings where the flow and mixture of fuel, oxygen, and heat is consistent (Ballal & Lefebvre, 1975), the variability of these factors contributes to flame turbulence. This leads to a situation where a fire that has a consistent average intensity over a period of time can have flickering, puffing, and noise that contributes to the fire perceptually varying moment to moment. Understanding how precisely fire intensity can be perceived across the lifespan is important to predicting human behavior in fire. When encountered during an incident, occupants can use the perceptual characteristics of fire, including the visual and olfactory cues from flames and smoke, to determine whether a fire is indeed present, the risk posed by it, and inform the potential actions that can be taken (Kuligowski, 2013). As such, identifying the extent to which children and adults can detect differences in fire intensity, and the underlying cognitive mechanisms, can inform fire safety measures.

Behavioral evidence of ratio effects with adults suggests that visual fire intensity estimates may be supported by approximate magnitude representations. Bonny and Milke (2023) developed a fire comparison task, based on those used with non-symbolic quantity research, to evaluate whether ratio effects were observed with fire intensity. One experiment focused on adults’ ability to judge which of two videos displayed a fire that was more intense. The videos were developed using simulated fires generated to numerically model fire dynamics (Fire Dynamics Simulator, FDS, McGrattan et al., 2021). The fires were systematically controlled to vary in intensity (heat release rate, HRR) by a specific ratio, ranging from smaller to larger. Ratio effects were indeed observed with estimated fire intensity *w* values between 0.03 and 0.09 for adults (Bonny & Milke, 2023). This placed fire intensity precision within a similar range as prior observations with non-symbolic number and spatial extent estimates with adults (Odic, 2018). This research suggests that, at least with an adult sample, relatively small differences in fire intensity could be accurately discerned using visual cues displayed over time. The similar range of *w* values to other types of non-symbolic quantities suggests that fire intensity may share other characteristics of approximate magnitude representations reported in prior research. With past research investigating the precision of fire intensity perception with adults, a remaining gap is the precision of children. Given the variability in visual cues correlated with fire intensity, it is unclear whether young children can reliably detect differences during comparison tasks. Observing a developmental trend similar to other quantities would provide additional evidence that estimates of fire intensity are supported by approximate magnitude representations, potentially with similar cognitive mechanisms.

### 1.3. Present Study

The goal of the present study was to examine children’s precision when comparing fires of different intensities using visual cues. Developmental research that has examined non-symbolic quantity perception was referenced when determining age groups to investigate. Specifically, the precision of numerical and spatial information undergoes the most pronounced age-related improvements between three and six years of age (Halberda & Feigenson, 2008; Odic, 2018). A child-friendly comparison task was developed to present fire intensity comparisons to young children. Similar to Bonny and Milke (2023) and prior non-symbolic quantity research, the ratio between fire intensities in two simultaneously displayed videos was varied from smaller (larger intensity divided by smaller intensity) to larger, with the accuracy of judgments recorded. In addition to children, the parents of child participants were administered the same task to provide an adult comparison. In this way, the present study provides a novel investigation of age-related differences in fire intensity perception. The hypotheses that were examined included children’s performance during the comparison task would vary by ratio and the precision of fire intensity perception would be significantly better with older children. This would suggest that fire intensity follows a similar developmental trajectory as other non-symbolic quantities.

## 2. Materials and Methods

### 2.1. Open Practices Statement

Data and materials used in this research are available in an online repository (https://osf.io/gpv8k/). No experiments were preregistered.

### 2.2. Participants

A total of 80 children and 100 parents completed the study. Families were recruited from participant panels, the online platform CloudResearch Connect (54 children, 66 parents) and Sago (26 children, 34 parents). Parents self-reported demographic characteristics as well as socioeconomic status (SES; see Supplementary Material for measure used); responses indicated that families varied in demographics (race: *N* White = 69, *N* Black or African American = 27, *N* Asian = 4; see Table 1). Parents received a monetary incentive for completing each part of the study at a minimum rate of $15 per hour. Parents provided informed consent and children provided assent as part of the study protocol in accordance with the Declaration of Helsinki and approved by the Institutional Review Board of Morgan State University (#22/02-0011).

Participant recruitment occurred over several sessions (experiment procedure was similar for Connect and Sago platforms). Individuals that self-reported being a parent of a child within the target age range were invited to complete a prescreen survey. Participants that responded to the survey as having an internet-connected computer with a webcam, a child within the target age range, being willing to video record their child completing the task, not having experienced a past fire incident, and willing to continue with the study were invited to the next session (167 out of 221 responses eligible). During this next session, parents were asked to complete the fire comparison task themselves and self-report demographic characteristics; those that completed the parent task were invited to the third session (100 eligible). During the third session, parents assisted their children to complete the fire comparison task. Webcam recordings were taken during this session to visually check, post hoc, whether the child was likely within the target age range. All participants included in data analyses passed this check.

### 2.3. Materials

All tasks were coded using the jsPsych JavaScript library (de Leeuw, 2015) and hosted online via a JATOS server (Lange et al., 2015) via the DigitalOcean platform (DigitalOcean Holdings, Inc.).

Participants were presented with multiple video clips displaying simulated fires. The videos were based on those used in Bonny and Milke (2023). Videos displayed numerical simulations of fires, generated with Fire Dynamics Simulator (FDS) software (version 6.7.6). The numerical results were visually rendered using PyroSim (Thunderhead Engineering, version 2021) to display fires burning at different intensities. Using FDS, the source of the fires was a square burner that had a consistent width during the videos. The intensity was varied by adjusting the heat release rate (HRR) per unit area of the burner. Within each video, fires displayed dynamic visual characteristics including flickering of flames, production of smoke, and changes in color and shape. Pairs of simulated fires were selected to generate videos that displayed a specific ratio difference in HRR. Each video was eight seconds in duration (30 frames per second).

**Figure 1:**
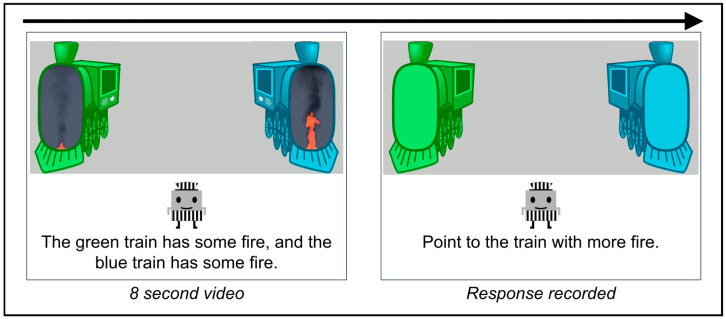
Trial procedure for the fire comparison task.

To present fires in a child-friendly format, each fire was embedded within a cartoon train engine (Figure 1). The trains were located at opposing ends of the horizontal video (950 by 500 pixels) and were color-coded (blue, green). Each fire was scaled and superimposed over the front of the engine compartment (132 by 256 pixels). This template was used to depict the fires as powering the trains.

### 2.4. Task Procedure

Parents and children received similar fire comparison tasks with the main differences being in the cover story, ratios presented, and response methods. Participants were informed that they would view several train engines that used fire to make them move. The instructions emphasized the intensity of fires: trains with more fire would move faster. Participants were told that they would see several pairs of blue and green trains and that they would need to determine which train had more fire. All instructions were provided by a cartoon character that appeared at the center of the screen in both via audio recordings and on-screen text captions. The character then demonstrated how to complete a trial, twice. Afterwards, participants were then presented with two practice trials with the correct answer identified after a response was made (one large ratio was used for these trials, 12:1). They were next presented with the test trials.

For each trial, participants were first presented with an attention screen that displayed an animation of a bouncing ball in the center of the scene. Participants were prompted by the cartoon character to press a ‘start’ button when ready. Afterwards, the fire video was displayed: each train was presented with the fire covered for 500 ms followed by the overlaid fires displayed for 8 s and a 500 ms presentation of the trains with the fire covered once again. Next, images of each train (without fire) were displayed as buttons and the character prompted the participant to select the train that had more fire. For the child task, the child was asked to either select the train or to point to the train, with the parent making the response. Instructions specified that parents should only record the response the child made and to encourage a response but not tell the child which train to select. Webcam recordings were further used to discourage parents from telling their child which train to select. These approaches follow recommendations for conducting online research with parents and children to minimize parents telling their child how to respond during a study (Shields et al., 2021). After a response was made, participants were provided with a reward animation (e.g., shining sun) and the next trial was presented.

### 2.5. Experiment Design

A within-subjects factor of ratio was used for the fire comparison task to examine performance. For children, this included (from smallest to largest; 5 levels): 1.05, 1.20, 2.00, 4.00, 8.00; for parents, this included additional ratios of 1.11 and 1.50 and the omission of the 4.00 ratio (6 levels)^1^. Four trials were presented for each ratio, with the side displaying more intense fire counterbalanced across the task. Ratios were selected based on prior research with adults suggesting that some individuals require a greater difference in fire intensities to provide accurate judgments (Bonny & Milke, 2023). As a result, some selected ratios were larger than those typically used in non-symbolic number and spatial extent comparison tasks.

Participant age was analyzed as a subject variable in two ways. For task performance analyses, age was entered as a continuous variable (years old). For comparing *w* fractions to prior research, age was categorized into corresponding bins: 3-, 4-, 5-, 6- year-olds and adults.

## 3. Results

Data were analyzed using R (version 4.4.1) and corresponding packages (lme4, version 1.1-35.5, Bates et al., 2015; lmerTest, version 3.1-3, Kuznetsova et al., 2017; car, version 3.1-2, Fox & Weisberg, 2019; emmeans, version 1.10.4, Lenth et al., 2022; ggplot, version 3.5.1, Wickham, 2016); tests were two-tailed (α = 0.05). Continuous factors were scaled and centered when entered as predictors and post hoc comparisons were adjusted using Holm corrections.

### 3.1. Response Accuracy

A set of binomial tests (corrected for multiple comparisons) examined which ratios had significantly more correct responses for each age group. For 3-year-olds, performance was above chance for 2.00, 4.00, and 8.00 ratios (adj. *p*s < 0.001). For 4-, 5-, and 6- year-olds, above chance performance also included the 1.20 ratio (adj. *p*s < 0.05). For adults, performance at the additional ratios of 1.11 and 1.50 (4.00 ratio was omitted) were above chance (adj. *p*s < 0.001) but, similar to children, performance at the 1.05 ratio was at chance. Overall, these results suggested that children and adults were able to detect differences in fire intensity at the largest ratios.

**Figure 2:**
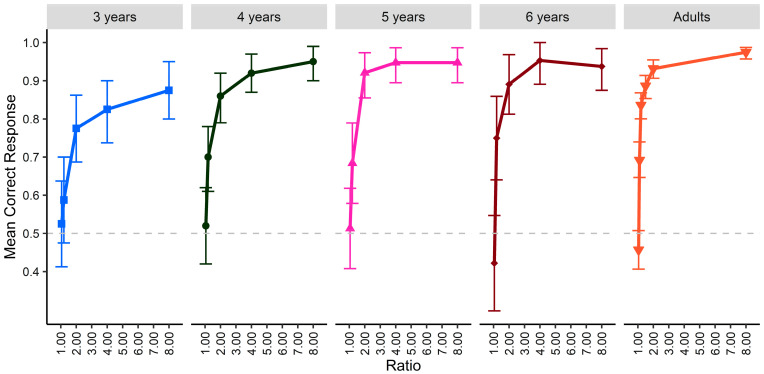
Performance of children and adults by age group on the comparison task for each ratio. Error bars indicate 95% confidence intervals.

A mixed model logistic regression (random intercept for participant) was used to analyze whether correct responses (1; 0 = incorrect) varied by ratio and participant age (both were entered as continuous variables, scaled and centered). A significant interaction between ratio and age was observed, coefficient = 0.45 (0.16), *z* = 2.86, *p* = 0.004, *odds ratio* (*OR*) = 1.57, along with significant main effects of ratio, coefficient = 1.59, (0.18) *z* = 8.83, *p* < 0.001, *OR* = 4.93, and age, coefficient = 0.47 (0.10), *z* = 4.59, *p* < 0.001, *OR* = 1.60. A post hoc analysis run using the ‘simr’ R package (100 simulations; version 1.0.7, Green & MacLeod, 2015) indicated that the model was sufficiently powered to detect the observed effect sizes (ratio: power = 1.00, 95% confidence interval = 0.96 to 1.00; age: power = 1.00, 95% confidence interval = 0.96 to 1.00; ratio by age: power = 0.97, 95% confidence interval = 0.91 to 0.99). Post hoc tests examined whether the general age effect of higher probability for correct responses with older participants was observed at each ratio presented to children. The effect of age was significant for each ratio (adj. *p*s < 0.05); the impact of age increased with ratio, with a greater disparity in the probability of correct responses for younger children versus adults with larger ratios (Figure 2). This aligns with the hypothesis that performance on the fire comparison task would increase with age and ratio.

### 3.2. Weber Fraction (w) Estimates

To allow for comparisons to prior research, *w* values were estimated for each age group. The approach used the sigmoid function from Odic (2018) which included *w* and a lapse coefficient (*l*), to account for guessing (see Supplementary Material). The estimated *w* values and lapse rates were as follows: 3-year-olds, *w* = 0.43 (standard error, *SE* = 0.18), *l* = 0.25 (*SE* = 0.14); 4-year-olds, *w* = 0.25 (*SE* = 0.06), *l* = 0.15 (*SE* = 0.05); 5-year-olds, *w* = 0.25 (*SE* = 0.06), *l* = 0.10 (*SE* = 0.06); 6-year-olds, *w* = 0.22 (*SE* = 0.06), *l* = 0.13 (*SE* = 0.06); adults, *w* = 0.14 (*SE* = 0.01), *l* = 0.11 (*SE* = 0.02). These were in line with the values reported in prior research with children and adults for number (Halberda & Feigenson, 2008; Odic, 2018), area (Odic et al., 2013), density, and time (Odic, 2018). Similar to the performance analysis, *w* values decreased with age, indicating greater precision with older individuals.

### 3.3. Developmental Trend Analysis

**Figure 3:**
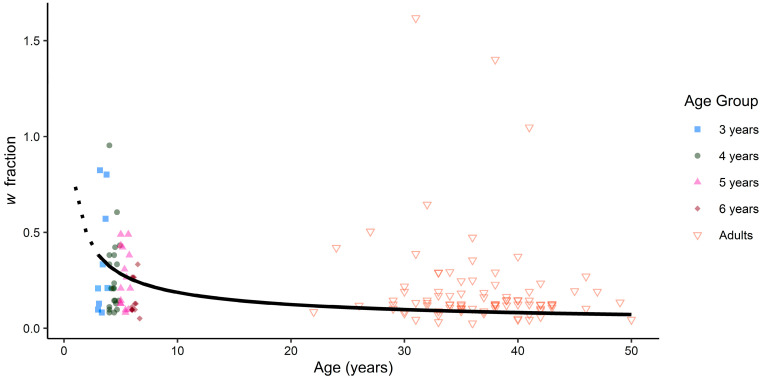
Fitted power curve with estimated *w* fractions across ages (dashed line is for estimates at ages below the tested age range). Points indicate observed participant *w* fractions.

A power model, adapted from Halberda and Feigenson (2008) and Odic et al. (2013), was used to examine the growth rate of fire intensity precision. In the power model (*w* = *a* × *age ^b^*), coefficient *a* is a scaling factor and indicates the *w* value when age equals one year with coefficient *b* influencing the rate of change. Lower values of both coefficients indicate more precise *w* values at an earlier age. However, lower values of coefficient *b* indicate more rapid age-related changes in *w*. Age group *w* values from the sigmoid function that included lapse rate were entered with the mean age (in years) into the model (see Supplementary Material). Individual *w* values were estimated to illustrate the alignment of the power model with individual performance (*w* values were fit for 146 participants, convergence failed for the remaining participants; see Figure 3). The estimated *a* coefficient was 0.74 (*SE* = 0.34) with a *b* coefficient of −0.60 (*SE* = 0.29).

## 4. Discussion

The goal of the present research was to investigate the visual perception of fire intensity across early childhood. To do so, children and adults were presented with a comparison task where the ratio difference in the intensities of two fires was varied from smaller to larger. The modeling approaches of prior developmental research with non-symbolic quantity perception were applied to estimate the precision of fire intensity perception at different ages. Results indicated that young children could detect differences in fire intensity and older children were more adept at the task. This provides initial evidence that young children can use visual cues to differentiate the intensities of controlled fires. The estimated developmental trend further suggests that visual fire intensity is supported by approximate magnitude representations.

### 4.1. Evidence of Child Fire Perception and Age-Related Differences

The presence of ratio effects indicated that children in the youngest age group were able to perceive visual cues of fire intensity. Comparisons to chance revealed that children could reliably detect which fire was greater in intensity on trials with larger ratios. In addition, the presence of a significant interaction effect indicated that performance with larger ratios was greater for older age groups. Ratio effects in behavioral performance, specifically response accuracy, have been used as evidence of approximate non-symbolic quantity representations in early childhood (Halberda & Feigenson, 2008). The ratio effects in the present study provide support for the hypothesis that children would be able to use approximate representations to detect differences in fire intensity.

Improvements in performance suggested that approximate representations of fire intensity were more precise with older age groups. Binomial tests and logistic regression results indicated that older children and adults were more likely to select the fire that was more intense, with this age effect more pronounced for larger ratios. A similar age-related effect was observed with estimated *w* values: they were smaller, indicating greater precision, for older children and adults. With regard to *w* values, pronounced improvements in precision were observed within the age range of children in the present study, three to six years. This aligns with prior research that has also observed significant improvements in non-symbolic quantity *w* fractions in early childhood (Bonny & Lourenco, 2013, 2015; Halberda & Feigenson, 2008; Odic, 2018). For adults, the *w* values observed in the present study were higher than previously observed for fire intensity comparisons in Bonny and Milke (2023). This is likely due to differences in the experiment procedure, specifically the inclusion of corrective feedback in the prior study by Bonny and Milke (2023) and lack thereof in the present research (participants received a reward animation regardless of whether the response was correct). This aligns with evidence that providing corrective feedback may improve performance on non-symbolic comparison tasks involving number (DeWind & Brannon, 2012; but see Lindskog et al., 2013). It suggests that, at least with adults, corrective feedback may impact the extent to which individuals can use visual cues to compare fire intensities. Overall, the results of the present study provide initial evidence that mental representations corresponding to visual fire intensity improve in precision over early childhood.

### 4.2. Developmental Trend Comparisons of Fire and Prior Estimates of Non-Symbolic Quantity Precision

The estimated precision of fire intensity representations aligns with developmental trends observed previously with non-symbolic quantities, with some deviations. With regard to 3-year-olds, estimates of *w* for fire intensity (*w* = 0.42) were less precise than prior estimates of length (*w* = 0.34), and were more precise than estimates of time, number, area, and density (*w* values = 1.27, 0.98, 0.86, 0.72, respectively; Odic, 2018). This pattern in *w* values with respect to fire was consistent for length, density, and time across 5-year-olds and adults; number *w* was comparable to fire intensity for adults, and area *w* was comparable for 5-year-olds, becoming more precise than fire intensity for adults. When comparing the fitted power curve model to Odic et al. (2013) for children and adults included in their study, the estimated exponent coefficient *b* for fire intensity (−0.60) was greater than number (−0.76) and area (−0.84; this pattern held when estimating *w* without a lapse rate, see Supplementary Material). In summary, the rate of age-related change in fire intensity precision was slower than observed for number and area in prior research. A limitation to these comparisons is the use of *w* estimates across studies that utilized different methods and age ranges. Nonetheless, these estimates indicate that the visual perception of fire intensity displays some of the developmental characteristics previously observed with density and time perception, with a slower rate of improvement compared to area and number.

Similarities in developmental trends raise questions as to whether the mental processes underlying fire intensity perception across development are shared with those of other quantities. Prior work has contrasted the predictions of a general magnitude system perspective, where a common representation underlies all non-symbolic quantities (Bueti & Walsh, 2009; Dakin et al., 2011; Walsh, 2003), against a multi-system perspective, where distinct representations correspond to specific quantities (Burr & Ross, 2008; Odic, 2018). In the present comparison of prior age-related trends in non-symbolic quantities with fire intensity precision, there was mixed evidence. Although some similarities for developmental trend parameters were observed, these were inconsistent: the quantities similar to fire intensity for some parameters were different for others. This raises the question of whether children’s perceptions of fire intensity are supported by a single or several approximate magnitude representations. Fire intensity may be directly processed via perceptual cues as a mental representation, similar to spatial extent or duration. This could be in line with the shared view, with the resulting magnitude representation for fire intensity overlapping with other quantities, or discrete view, with the approximate representation being independent and specific to fire intensity. The inconsistencies in developmental trend parameters may point to judgments of fire intensity relying on multiple approximate magnitude representations. This would align with observed correlations with fire intensity in the real-world. During combustion, multiple cues are strongly associated with the intensity of the fire, including the visual cues of size, color, flickering, soot production, and smoke opacity (Quintiere, 2006). In summary, more intense fires have larger flames (spatial extent), thicker smoke (luminance), and produce more heat (temperature). This perspective would align with the distinct view of magnitude representations that has focused on non-symbolic number (Odic, 2018). Specifically, it has been argued that non-numerical quantities that are correlated with number and embedded within the same stimulus, such as spatial extent, can inhibit or facilitate performance, but do so via alignment as a separate representation encoded in parallel. As applied to fire intensity in the present study, distinct representations of spatial extent and luminance may have been estimated, for example, from the simulated fires and simultaneously contributed to judgment performance. Indeed, prior research that prompted adults to report what they used to visually discriminate between fire intensities observed that flame size and height were the cues most frequently reported (Bonny & Milke, 2023). Children, like adults, may also attend to these cues when estimating fire intensity. However, this hypothesis will need to be investigated in future research (see Supplementary Materials for a descriptive analysis of spatial extent and visual characteristics of stimuli on performance).

The inconsistencies in the developmental trend parameters may reflect differences in the relative attention to specific quantities, which change with age. Similar variations have been proposed with regard to the salience, and biasing effect, of spatial extent and number across the lifespan (Aulet & Lourenco, 2021; Starr et al., 2017). Disentangling the influence and salience of quantities correlated with fire intensity across early childhood can further address the development of approximate fire intensity representations.

### 4.3. Limitations and Future Directions

The context in which fire intensity stimuli were presented lacked ecological realism. Although simulation software was used to create videos of realistically behaving fires, they were presented in an artificial manner using train-related scenery. In addition, the fires were confined to a single location with a relatively small footprint. Taking a similar approach to prior non-symbolic number approximation research (Odic & Oppenheimer, 2023), future studies can utilize stimuli where fires are situated within different types of ecologically relevant contexts.

The present research provides evidence for the development of fire intensity perception following similar patterns as approximate quantity representations. However, future research should explicitly compare children’s *w* values for fire intensity to other types of non-symbolic quantities. The present study was limited to a fire intensity comparison task. By including the same experiment procedure with multiple quantities, future studies can directly test whether fire intensity precision overlaps or deviates from that of length, density, and time. Following prior research, the within-subjects design can be used to examine whether individual differences in children’s fire intensity precision correlate with those of other quantities (e.g., Lourenco & Bonny, 2017). This can allow for a greater understanding of how children and adults perceive the intensity of fire using visual cues. In addition, studies examining connections with non-symbolic number precision and the influence of spatial extent information, have investigated connections with individual differences in inhibitory control (Fuhs & McNeil, 2013; Gilmore et al., 2013). Taking a similar approach, connections with children’s inhibition capabilities can be assessed to evaluate how the relative salience of spatial extent and luminance when judging fire intensity may be connected to executive function processes. By utilizing previous research methods used with approximate representations of number, spatial extent, and time, the cognitive mechanisms underlying children’s perception of fire intensity can be investigated.

The methods of the present study can be used to examine the extent to which an approximate magnitude system more broadly encompasses dynamic quantities. The present study was limited to fire intensity with evidence that visual perception of dynamic quantities can be supported by approximate representations. Similar developmental trends may be observed for these types of ecologically relevant quantities, such as water flow. However, given the cultural relevance and inclusion of fire in daily activities, it is possible that other types of ecologically relevant quantities may have less precision and prolonged age-related improvements in *w* fractions. Future research can investigate how the approximate magnitude system applies other types of dynamic, ecological quantities.

## Figures and Tables

**Table 1:** Demographics of participants by age group (in years).

Demographic	3-Year-Olds	4-Year-Olds	5-Year-Olds	6-Year-Olds	Adults
*N*	24	23	19	14	100
*N* female	11	9	11	9	75
Age Mean	3.52	4.41	5.37	6.17	36.28
Age Min	3.00	4.00	5.00	6.00	22.00
Age Max	3.92	4.92	5.83	6.67	50.00
SES ^a^ Mean	4.79	5.43	4.68	5.92	5.07
SES ^a^ Min	1.00	1.00	1.00	2.00	1.00
SES ^a^ Max	7.00	8.00	7.00	9.00	9.00

^a^ SES = socioeconomic status.

## Data Availability

Data and materials used in this research are available in an online repository (https://osf.io/gpv8k/).

## Note

1 One parent reported that they mistakenly completed the child task; their data was included for analysis with the adult age group but omitted from Figure 2.

## Supplementary Materials

The following supporting information can be downloaded at https://www.mdpi.com/article/10.3390/bs15101397/s1, Figure S1: Image of the social ladder provided to parents to indicate socioeconomic status; Figure S2: Example of a fire region (left) with a mask applied (right) to a video frame to estimate spatial metrics of flame regions (horizontal line indicates the maximum flame height); Table S1: Estimated Weber fractions (*w*) for each age group without including a lapse rate; Table S2: Pearson correlations between ratios for heat release rate (HRR) and extracted metrics for each video; Table S3: Logistic regression model performance for heat release rate (HRR) and video metric ratios (Adler et al., 2000; Bonny & Milke, 2023; Brannon et al., 2006; Donderi, 2006; Kramer et al., 2011; Lourenco & Longo, 2010; Odic, 2018; Piantadosi, 2016; Quintiere, 2006; Sanford & Halberda, 2024; Stamps, 2002).

## Abbreviations

The following abbreviations are used in this manuscript:

SES	Socioeconomic status
HRR	Heat release rate
OR	Odds ratio
*w*	Weber fraction
