# Supplementary material for: Seeing Flames, Perceiving Quantity: Approximations of Fire Intensity Across Development
Source: Behav Sci (Basel). 2025 Oct 15;15(10):1397. doi: 10.3390/bs15101397 (PMC12561452; doi:10.3390/bs15101397)
Supplement: Supplementary file 1 [file behavsci-15-01397-s001.zip › behavsci-3746057-supplementary.pdf]

Seeing Flames, Perceiving Quantity: Approximations of Fire Intensity Across Development

Supplementary Material

Method

Socioeconomic Status Ladder Question

Parents were asked to report their socioeconomic status (SES) using a social ladder question (Adler et al., 2000). For this question, parents were provided with the following prompt along with an image of a social ladder (Figure S 1); "Think of this as representing where people stand in society. At the top of the ladder are the people who are best off, those who have the most money, most education and the best jobs. At the bottom are the people who are worst off, who have the least money, least education and the worst jobs or no job. The higher up you are on this ladder, the closer you are to people at the very top and the lower you are, the closer you are to the bottom. Where would you put yourself on the ladder? Choose the number whose position best represents where you would be on this ladder."

Figure S 1

Image of the social ladder provided to parents to indicate socioeconomic status.

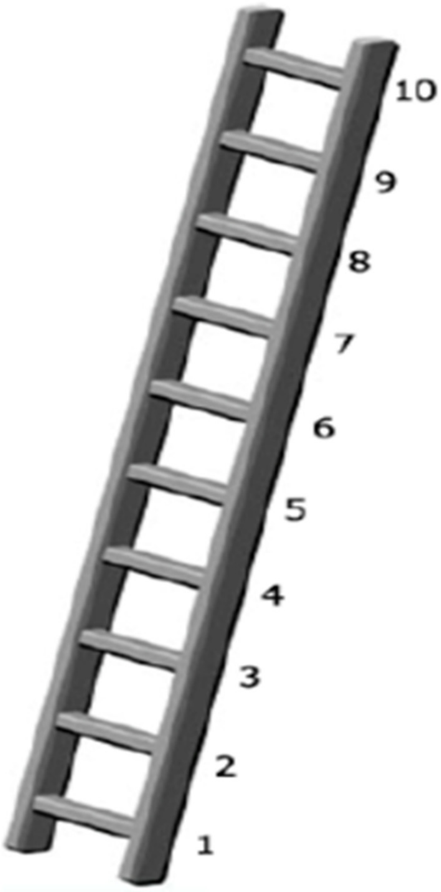

Results

Weber fraction (w) Calculation

For each age group, the model was fit to responses made by all participants using the '*nls*' *R* function (starting parameter estimates were selected from the fitted coefficients for density for 5-year-olds from (Odic, 2018): w = 0.40, *l* = 0.05; Equation 1, in *R* formula format). This model was used to allow for direct comparisons of *w* values of fire intensity to those of other non-symbolic magnitudes reported in Odic (2018), which also included the lapse rate parameter (*l*).

$$correct \sim (1 - l) * pnorm((ratio - 1)/(w * sqrt(1 + ratio^{\wedge}2))) + l/2 \quad (1)$$

A second set of w values were fitted to allow for better alignment when comparing developmental trends to Odic and colleagues (2013) and Halberda and Feigenson (2008). Across these studies, the common method used to estimate *w* values for modeling developmental trends was to not include a lapse rate coefficient. To this end, the approach developed by Piantadosi (2016) to model *w* values was used to estimate *w* values for each age group via the presented *R* function (in *R* formula format) where *ai* and *bi* are the pairs of values (fire intensities), *ri* is whether the response was correct (1) or incorrect (0), and *W* is the Weber fraction parameter:

```
optimize (function (W) {
  pcorrect <- pnorm(abs(ai-bi)/(W * sqrt (ai **2+bi**2)))
  -log (W) + sum(log (ifelse (ri, pcorrect, 1-pcorrect)))
}, maximum=TRUE, interval=c (0, 3))
```

The fitted *w* values for the model that lacked the lapse rate were higher overall than when the lapse rate was included (see Table S1). However, the general age-related trend was relatively consistent, with smaller *w* values for older compared to younger age groups.

Table S1

Estimated Weber fractions (w) for each age group without including a lapse rate.

Age Group	*w*
3 years	0.72
4 years	0.48
5 years	0.43
6 years	0.45
Adults	0.29

Developmental Trend Calculation

A power curve model was adapted from Odic and colleagues (2013) to fit participant *w* values across age (in years). Model parameters were estimated using the '*nls'* R function. To fit the model, the fitted *w* values for each age group (see Section 3.2 in manuscript) were entered along with the mean age (in years) for each age group (3-year-olds, 4-year-olds, 5-year-olds, 6-year-olds, adults; see Table 1 in manuscript). Starting parameter estimates were selected from the fitted coefficients for area (when estimated infant *w* values were omitted) from Odic et al. (2013): a = 0.95, b = -0.84; Equation 2, in *R* formula format).

$$w \sim a * age \char`^ b \quad (2)$$

To allow for further comparison with Odic and colleagues (2013), a second power model was fitted using the *w* values estimated without the inclusion of a lapse rate (Table S1). The

fitted coefficient *a* (0.99, *SE* = .30) and fitted coefficient *b* (-0.40, *SE* = 0.18) were greater compared to the power model fitted to lapse-included *w* values.

Video Flame Metrics and Performance

A descriptive analysis of fire videos explored how visual characteristics may have contributed to performance during the judgment task. Several characteristics are associated with heat release rate (HRR) including the height and pulsation of the flames (Quintiere, 2006) as well as the color of the flames and smoke (Bonny & Milke, 2023). In addition, prior studies investigating non-symbolic quantity comparison judgments have focused on the impact of spatial quantities on performance, including total surface area, total perimeter, and convex hull (Brannon et al., 2006; Lourenco & Longo, 2010; Sanford & Halberda, 2024). Based on this research, the following metrics were extracted from the videos: number of flame regions (e.g., base flame and detached flame puff = 2), total area encompassed by flame regions, total perimeter of flame regions, area of convex hull of flame regions, maximum point height of flames, average lightness of the entire frame (based on HSL color model), and Shannon's entropy of color (red, green, blue) of the entire frame. Of these metrics, entropy has been less utilized in prior quantity perception research. However, this was selected to both accompany lightness and to capture ordinal differences in the amount of complexity, or randomness, in the colors of rendered smoke and flames during the videos. Although luminance and brightness have been used in prior non-symbolic quantity research (Kramer et al., 2011), due to participants using different devices and monitors, a similar metric was not available for analysis. Instead, the mean 'lightness' channel values of the images (when in HSL) were selected as an approximation. Entropy was used in an attempt to supplement lightness with a device-agnostic statistic that could capture some of the image features. Indeed, prior studies have used Shannon's entropy as

an indicator of diversity and complexity in images (Donderi, 2006; Stamps III, 2002). With turbulence, opacity, color, and pulsation being observed to correlate with HRR in pyrolysis research, entropy was used as an indicator of these characteristics.

All calculations of metrics were conducted in Python using the OpenCV and numpy libraries. Metrics were calculated for each fire in each video frame (region containing the fire was 254-pixels in height and 129-pixels in width). Total entropy was calculated by applying Shannon's entropy equation to each channel (RGB) and summing the values: $H = -\Sigma (p_i \times \log_2(p_i))$, where p_i is the probability of i-th pixel intensity value (e.g., how often the value 128 appears in the red channel) and n is the number of unique pixel values (256; higher values indicated higher complexity). For spatial metrics, a mask was applied to select the flame regions; the cutoff values for the mask were visually estimated (lower RGB = 80, 40, 0; upper RGB = 255, 255, 100). A contour function was then applied to the resulting regions identified as flames to extract spatial metrics in pixels (note: whereas OpenCV sets the origin of an image in the top-left corner, maximum height was transformed such that the origin was in the bottom-left corner, with greater values indicating higher flames;

Figure S 2). To estimate the video metric, the mean values across all frames were calculated. Similar to the use of HRR in the main analyses, the ratio of the more intense to less intense fire was calculated for each video metric (higher ratios indicated the metric was greater in the more intense fire).

Figure S 2

Example of a fire region (left) with a mask applied (right) to a video frame to estimate spatial metrics of flame regions (horizontal line indicates the maximum flame height).

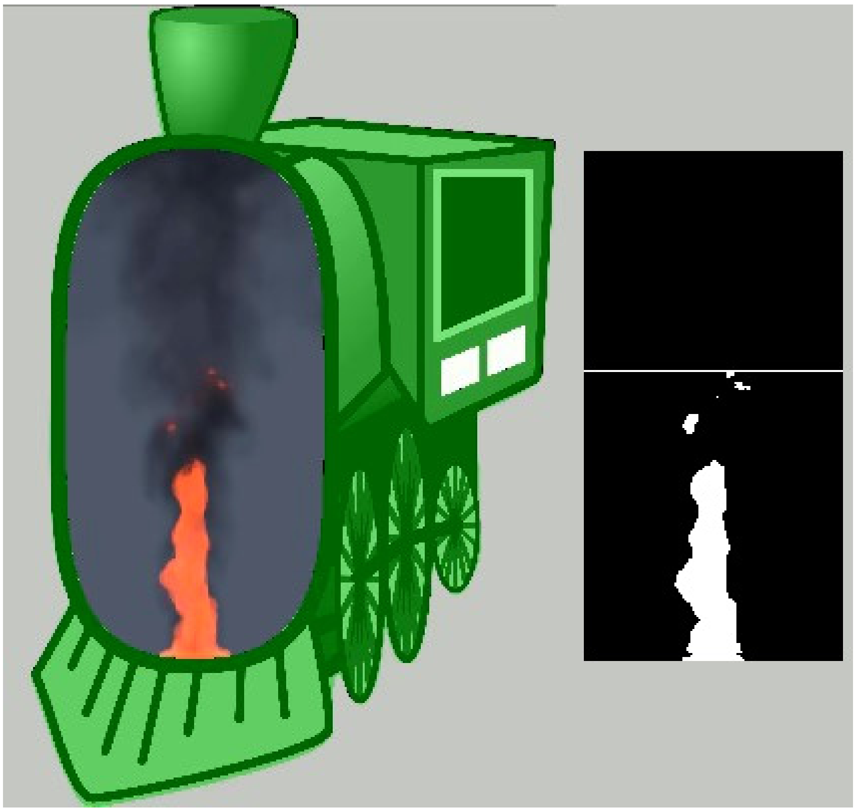

Table S 2

Pearson correlations between ratios for heat release rate (HRR) and extracted metrics for each video.

	HRR	*N* Flame Regions	Area	Perimeter	Convex Hull	Entropy	Height
***N* Flame Regions**	0.936***	—	—	—	—	—	—
**Area**	0.995***	0.908***	—	—	—	—	—
**Perimeter**	0.991***	0.954***	0.985***	—	—	—	—
**Convex Hull**	0.997***	0.920***	0.999***	0.988***	—	—	—
**Entropy**	0.968***	0.946***	0.959***	0.989***	0.962***	—	—
**Height**	0.990***	0.912***	0.996***	0.990***	0.995***	0.972***	—
**Lightness**	-0.940***	-0.946***	-0.927***	-0.971***	-0.932***	-0.991***	-0.943***

As expected, strong correlations were observed between metric ratios (Table S2). Due to multicollinearity, separate logistic regression models predicting response accuracy (random intercept for participant) were fitted for each metric and compared (metrics were scaled and centered; variance inflation factors, VIFs, when entered simultaneously as predictors were greater than 39). To provide some comparison, fit statistics were estimated, including *AIC* and marginal R^2 (estimated using the 'MuMIn' *R* package). Overall, fit statistics were similar across video metrics, with perimeter and lightness having slightly better marginal R^2 and *AIC*, respectively (Table S3). However, the model with HRR, in contrast, outperformed all video metrics.

Table S 3

Logistic regression model performance for heat release rate (HRR) and video metric ratios.

Metric	Coefficient	Coefficient Standard Error	*AIC*	Marginal R^2
HRR	1.20	.091	3738	.286
N Flame Regions	.719	.053	3859	.127
Area	.757	.056	3852	.139
Perimeter	.806	.057	3820	.155
Convex Hull	.747	.056	3858	.136
Entropy	.796	.054	3811	.151
Height	.797	.056	3823	.152
Lightness	-.777	.051	3805	.145

References

Adler, N. E., Epel, E. S., Castellazzo, G., & Ickovics, J. R. (2000). Relationship of subjective and objective social status with psychological and physiological functioning: Preliminary data in healthy, White women. *Health Psychology*, *19*(6), 586–592. https://doi.org/10.1037/0278-6133.19.6.586

Bonny, J. W., & Milke, J. A. (2023). Precision of Visual Perception of Developing Fires. *Fire*, *6*(9), Article 9. https://doi.org/10.3390/fire6090328

Brannon, E. M., Lutz, D., & Cordes, S. (2006). The development of area discrimination and its implications for number representation in infancy. *Developmental Science*, *9*(6), F59-64. https://doi.org/10.1111/j.1467-7687.2006.00530.x

Donderi, D. C. (2006). Visual complexity: A review. *Psychological Bulletin*, *132*(1), 73–97. https://doi.org/10.1037/0033-2909.132.1.73

Halberda, J., & Feigenson, L. (2008). Developmental change in the acuity of the "Number Sense": The approximate number system in 3-, 4-, 5-, and 6-year-olds and adults. *Developmental Psychology*, *44*(5), 1457–1465. https://doi.org/10.1037/a0012682

Kramer, P., Bono, M. G. D., & Zorzi, M. (2011). Numerosity estimation in visual stimuli in the absence of luminance-based cues. *PloS ONE*, *6*(2), e17378. https://doi.org/10.1371/journal.pone.0017378

Lourenco, S. F., & Longo, M. R. (2010). General magnitude representation in human infants. *Psychological Science*, *21*(6), 873–881. https://doi.org/10.1177/0956797610370158

Odic, D. (2018). Children's intuitive sense of number develops independently of their perception of area, density, length, and time. *Developmental Science*, *21*(2), e12533. https://doi.org/10.1111/desc.12533

Odic, D., Libertus, M. E., Feigenson, L., & Halberda, J. (2013). Developmental change in the acuity of approximate number and area representations. *Developmental Psychology*, *49*(6), 1103–1112. https://doi.org/10.1037/a0029472

Piantadosi, S. T. (2016). Efficient estimation of Weber's W. *Behavior Research Methods*, *48*(1), 42–52. https://doi.org/10.3758/s13428-014-0558-8

Quintiere, J. G. (2006). *Fundamentals of Fire Phenomena*. Wiley.

Sanford, E. M., & Halberda, J. (2024). Non-numerical features fail to predict numerical performance in real-world stimuli. *Cognitive Development*, *69*, 101415. https://doi.org/10.1016/j.cogdev.2023.101415

Stamps III, A. E. (2002). Entropy, Visual Diversity, and Preference. *The Journal of General Psychology*, *129*(3), 300–320. https://doi.org/10.1080/00221300209602100
